# Landscape of Biomarkers and Pathologic Response in Rectal Cancer: Where We Stand?

**DOI:** 10.3390/cancers16234047

**Published:** 2024-12-02

**Authors:** Abrahams Ocanto, Macarena Teja, Francesco Amorelli, Felipe Couñago, Ariel Gomez Palacios, Diego Alcaraz, Ramón Cantero

**Affiliations:** 1 Department of Radiation Oncology, Hospital Universitario San Francisco de Asís, GenesisCare, 28002 Madrid, Spain; macarena.teja@genesiscare.es (M.T.); felipe.counago@genesiscare.es (F.C.); 2 Department of Radiation Oncology, Hospital Universitario Vithas La Milagrosa, GenesisCare, 28010 Madrid, Spain; 3 PhD Program in Medicine and Surgery, Doctoral School, Universidad Autónoma de Madrid, 28049 Madrid, Spain; 4 Department of Radiation Oncology, Hospital de la Santa Creu i Sant Pau, 08025 Barcelona, Spain; famorelli@santpau.cat; 5 Department of Medicine, School of Medicine, Health and Sport, European University of Madrid, 28670 Madrid, Spain; 6 Department of Radiation Oncology, Centro de Radioterapia Deán Funes, Córdoba 2869, Argentina; ariel.gomez@dfunes.com.ar; 7 Department of Medical Oncology, Hospital Universitario San Francisco de Asís, GenesisCare, 28002 Madrid, Spain; diego.alcaraz@genesiscare.es; 8 Colorectal Unit, Department of Surgery, La Paz University Hospital, 28046 Madrid, Spain; ramon.cantero@salud.madrid.org; 9 Department of Surgery, School of Medicine, Autonomous University of Madrid, 28029 Madrid, Spain

**Keywords:** rectal cancer, total neoadjuvant therapy, biomarkers

## Abstract

Colorectal cancer (CRC) is a neoplasm with a high prevalence worldwide, with a multimodal treatment that includes a combination of chemotherapy, radiotherapy, and surgery in locally advanced stages with acceptable pathological complete response (pCR) rates, this has improved with the introduction of total neoadjuvant therapy (TNT) reaching pCR rates up to 37% in compare with classic neoadjuvant treatment (NAT) where pCR rates of around 20–25% are achieved. However, the patient population that benefits most from this therapy has not been determined, and there is a lack of biomarkers that can predict the course of the disease. Multiple biomarkers have been studied, ranging from hematological and molecular markers by imaging technique and combinations of them, with contradictory results that prevent their use in routine clinical practice. In this review, we evaluate the most robust prognostic biomarkers to be used in clinical practice, highlighting their advantages and disadvantages and emphasizing biomarker combinations and their predictive value.

## Simple Summary

This manuscript aims to provide a comprehensive update on biomarkers in rectal cancer, focusing on both established and emerging markers. It highlights the most significant advancements in recent years, particularly those that align with the evolution of neoadjuvant treatment in this disease. Furthermore, it explores the relationship between biomarkers and pathological response, emphasizing their potential to serve as prognostic factors. Despite promising findings, many of these biomarkers have not yet been validated for routine clinical application. This review seeks to consolidate current knowledge, identify existing challenges, and propose future directions for research in this critical area of rectal cancer management.

## 1. Introduction

Colorectal cancer (CRC) is the leading cause of cancer-related death in men and the second leading cause in women [1]. Treatment has advanced in recent years, adopting a multimodal approach that includes surgery, chemotherapy (CT), and radiotherapy (RT) as mainstays [2]. This approach has reduced local recurrence rates, though high rates of distant metastasis persist in locally advanced stages [2,3]. The introduction of total neoadjuvant therapy (TNT), which incorporates early CT and preoperative chemoradiotherapy (CT-RT), has demonstrated additional benefits in eradicating micrometastases, making it one of the most widely accepted therapies today [2,3]. TNT achieves a pathological complete response (pCR) rate of 37%, theoretically reducing the risk of distant disease progression [3]. However, it remains unclear what factors influence the achievement of pCR and whether any modifiable factors could enhance outcomes following TNT.

## 2. Current State and Benefit of Neoadjuvant Treatment

Multiple studies on total neoadjuvant therapy (TNT) have explored the use of either short-course radiotherapy (SCRT) or long-course radiotherapy (LCRT) in combination with induction or consolidation chemotherapy (CT). This approach offers several notable advantages, including enhanced tumor reduction and high treatment compliance. The early introduction of CT not only allows for the assessment of chemosensitivity but also targets potential micrometastases [4]. Additionally, administering CT preoperatively may obviate the need for postoperative CT, thereby shortening the duration of ileostomy and significantly improving patients’ quality of life.

Since the introduction of neoadjuvant therapy for locally advanced rectal cancer (LARC), both sphincter preservation rates and local disease control have seen notable improvements [3]. Since then, numerous neoadjuvant treatment strategies have been developed with varying outcomes [5,6,7]. These strategies can be categorized as follows and encompass the different approaches within total neoadjuvant therapy (TNT):

### 2.1. Neoadjuvant Treatment (NAT)

#### 2.1.1. Short Course RT (SCRT)

Short-course radiotherapy (SCRT) consists of administering 25 Gy in 5 fractions with TME in the following 7 days, according to some studies, or, more recently, at 4–6 weeks [8]. In the Stockholm III clinical trial, the experimental arm that performed surgery between 4 and 6 weeks achieved a higher rate of downstaging at surgery compared to immediate surgery [8]. This treatment strategy reduces local recurrence by 2.4% when compared to surgery alone (8.2%) [9]. Subgroup analysis demonstrates a greater benefit in mid-to-lower rectal tumors, reaching 6-year local recurrence rates of 5.6% vs. 10.9% with exclusive surgery [10].

The meta-analysis published by Kasi et al. [2], which compared the efficacy of TNT vs. conventional CT-RT, demonstrated a higher likelihood of achieving pCR; however, surgical outcomes, including sphincter preservation and ileostomy complications, did not differ between the two populations.

According to some studies [11,12], patients who achieve pCR have a lower probability of local recurrence and a 5-year metastasis-free survival of 88.8% compared to patients who achieve a pathological partial response (pPR) of 74.9%, overall survival (OS) is around 87.6% at 5 years for patients who achieve pCR and 76.4% in those with objective pathological partial response (pPR).

#### 2.1.2. Long Course Chemoradiation (LCRT)

The long course chemoradiation (LCRT) has been applied since the results of the German Rectal Cancer Group [13] and consists of administering 50.40 Gy in 28 fractions in concomitance with radiosensitizing chemotherapy such as fluorouracil or capecitabine. After completing CT-RT, surgery is performed between 8 and 12 weeks. This study compared applying this treatment in neoadjuvant vs. adjuvant, demonstrating greater local control together with sphincter preservation in neoadjuvant [14,15].

When comparing both treatment strategies (SCRT vs. LCRT), similar oncologic outcomes have been demonstrated in terms of OS, local recurrence, and surgical complications [3].

Data from the German Rectal Trial (CAO/ARO/AIO-94 trial) [16] showed that 86% of patients who achieved pCR were disease-free at 5 years compared to 63% who achieved pPR, thus opening the debate on the importance of adjuvant CT vs. TNT with treatment intensification that can generate better long-term results, even in selected cases patients with pCR can enter the watch-and-wait (WW) strategy, which was introduced by Habr-Gama et al. [17] who objectified an OS of 97.7% and disease-free survival (DFS) of 84% after a decade of follow-up. A recent meta-analysis [18] found that patients who complete neoadjuvant treatment and achieve a radiological complete response have no difference in terms of local recurrence and cancer-related mortality than those patients who undergo surgery.

Both treatment strategies have been incorporated into routine practice according to the outcome of the following studies, with no predominance of one over the other. The phase II German Trial CAO/ARO/AIO-12 demonstrated that the use of 4 cycles of consolidation FOLFOX after LCRT can achieve a pCR rate of 25% [19]. Preliminary data from the OPRA trial that compared adding CT before or after LCRT incorporating the WW strategy in patients achieving pCR objectified similar DFS and disease-free metastases (DMS) in both arms but with a high organ preservation rate in patients managed with WW and receiving consolidation CT (58% vs. 43%) [20]. PRODIGE 23 Trial, randomized patients to receive six cycles of mFOLFIRINOX followed by LCRT, surgery, and three months of adjuvant chemotherapy versus standard therapy (LCRT + surgery + adjuvant chemotherapy). Patients in the experimental arm achieved pCR rates of 27.5% and 3-year DFS of 75.7% compared to 68% in the control arm [21]. RAPIDO Trial compared SCRT followed by consolidation CT (six cycles of CAPOX or nine cycles of FOLFOX) followed by surgery versus standard treatment (LCRT + surgery + adjuvant chemotherapy). The pCR rate in the experimental group was 27.7% versus 13.8% in the other arm, and disease-related treatment failure at 3 years was 23.7% compared to 30% in the control arm [22]. STELLAR Trial also used SCRT as the main strategy, comparing four cycles of CAPOX versus standard therapy with LCRT. Adjuvant CAPOX was included in both groups. The results showed a pCR of 22.5% in the experimental group compared to 12.6% in the control group and an OS of 86.5% in the experimental group versus 75.1% in the control group [23].

In view of the results of the TNT studies, the Timing Trial [24], which evaluates results after CT-RT and the number of cycles of mFOLFOX, is relevant, finding that patients who only completed CT-RT achieved pCR in 18%, those who completed two cycles 25%, those who completed four cycles 30% and those who reached six cycles 38%. All this is in agreement with those assessed in CAO/ARO/AIO-12 [19] and OPPRA Trial [20], which highlights that neoadjuvant CT is more beneficial than adjuvant. Data from the Janus Trial will also cooperate in this scenario (NCT05610163).

The advent of molecular targets has also been introduced in rectal cancer, adding PD-L1 blockers such as pembrolizumab in a neoadjuvant and adjuvant stage together with CT in the context of TNT (NRG-GI002); however, no difference in pCR rate was objectified (31.9% vs. 29.4%) [25]. Special mention should be made of the results of the study by A. Cercek et al. [26], where they added Dostarlimab, an anti-PD-1 antibody in patients with stage II-II rectal cancer who presented with mismatch repair-deficient (MRD), followed by QT-RT and surgery; however, patients who presented complete response with Dostarlimab were followed up without complementary therapies; 12 patients completed treatment and after a 6-month follow-up 100% had complete response assessed by MRI, PET-CT, rectoscopy, digital rectal examination and biopsy. Bando et al. [27] evaluated the combination of QT-RT followed by five cycles of nivolumab and surgery in patients with LARC and microsatellite instability (MSI); a pCR of 60% was achieved with very few immunomediated events.

The field of neoadjuvant therapy has advanced significantly and rapidly in recent years. However, as with other neoplastic diseases, reliable markers to predict tumor response are still lacking. In the case of rectal cancer, the ability to accurately predict tumor response is essential, as it could allow for the avoidance of aggressive and, in some cases, disfiguring surgeries that adversely affect patients’ quality of life. Among potential predictive markers, the most relevant and promising is the use of functional MRI sequences, followed by those identified in tumor biopsies and then blood-based markers (Table 1).

## 3. MRI as Prognostic Marker in Rectal Cancer

Magnetic resonance imaging (MRI) is a fundamental tool in the staging of rectal cancer, owing largely to high-resolution T2-weighted turbo spin echo (TSE) sequences acquired in multiple planes and aligned with tumor orientation, which significantly enhance diagnostic sensitivity. MRI provides critical information, allowing for the prediction of circumferential resection margin involvement, extramural invasion, venous invasion, and satellite tumor deposits. Its use following neoadjuvant therapy is also indispensable for evaluating treatment response and planning surgery. However, post-treatment edema, inflammation, and fibrosis can obscure accurate response assessment [28]. Identifying patients who are likely or unlikely to respond to neoadjuvant therapy—ideally from the onset of treatment—is of particular interest to avoid unnecessary toxicity [28]. Adding diffusion-weighted and perfusion-weighted sequences to rectal MRI enables a multiparametric assessment that enhances the precision of diagnostic evaluation:

Diffusion-weighted imaging (DWI): Useful sequence in case of changes in cell membrane integrity by tumor cells. Diffusion restriction can be seen in this sequence in the form of hyperintensity [28]. In a series of 45 patients diagnosed with rectal cancer, the sensitivity of detection of primary tumorigenesis increased from 82–84% to 93–96% when DWI was added to T2-weighted imaging [29]. Therefore, it is a useful sequence in the assessment of response after neoadjuvant therapy, and new publications suggest acquiring it in small FOV DWI mode [28].

Apparent diffusion coefficient (ADC): This value in the case of adenocarcinomas is significantly lower than that of the normal rectal wall [29]; a special case is that of mucinous adenocarcinomas in which the ADC values are higher in comparison with classic tubular adenocarcinomas [30]. There is controversy about the potential of this sequence to predict tumor response; some authors describe that there are no significant differences in patients who respond and those who do not respond after neoadjuvant treatment [31,32], while other authors point out that ADC can predict response to treatment early, one or two weeks after neoadjuvant treatment [33,34]. This inconsistency in results is primarily due to the limitations of retrospective studies and prospective cohorts with small sample sizes.

Perfusion MRI: This sequence allows the assessment of the microcirculation and vascular permeability in the tissue through a quantitative or semiquantitative assessment with some pharmacokinetic model that allows the assessment of the plasma flow (PF), plasma volume (PV), mean transit time (MTT), transfer constant (*K*^trans^), fractional extracellular leakage space (v_e)_, rate constant (*k*_ep_), initial slope, late slope, and others; and then be able to determine the capillary permeability that translates into transendothelial to contrast transport [28]. These parameters have been assessed primarily to aid in the detection of complete responses after neoadjuvant therapy. Martens et al. [35], in this setting, performed perfusion MRI at diagnosis and at 7–10 weeks after completion in 30 patients with rectal cancer with the aim of identifying any parameter with prognostic value of those analyzed they found that the late slope is the parameter that can be used prior to treatment and is the one that best correlates with tumor response after neoadjuvant treatment. Other studies focus on the fact that perfusion can predict tumor invasiveness [36].

MRI is a diagnostic tool with great potential when we add functional sequences; however, the parameters analyzed in the studies are not homogeneous, with different cut-off values, which makes it difficult to make a direct comparison and to establish a diagnostic protocol, which is why there are some studies with contradictory results in terms of prognosis of tumor response [28]. A special case is mucinous tumors where MRI is not able to distinguish cellular mucin (residual tumor) from acellular mucin (complete response) [37]. One way of trying to homogenize the assessment of tumor response after neoadjuvant treatment is the use of the MR tumor regression grade (mrTRG) criteria, with some discrepancies according to some studies due to the difficulty of their reproducibility [38,39]. These criteria consist of the evaluation of T2 weighted images and correlate them with the degree of tumor regression described in pathological anatomy [40,41], these criteria were born from the clinical trial MERCURY that demonstrated that the mrTRG criteria correlate with disease-free survival (DFS) and overall survival (OS) [42]:

- TRG1: Thin scar with no evidence of tumor.
- TRG2: Thick fibrosis with no clear residual tumor or minimal residual tumor.
- TRG3: Tumor and fibrosis (~50%).
- TRG4: Few areas of fibrosis and mostly tumors.
- TRG5: Unchanged or increased tumor without fibrosis.

This classification, together with morphologic assessment, makes it possible to recognize “good responders” after neoadjuvant treatment. It is known that a reduction > 60–80% correlates with a good prognosis [43], and if we add to this an assessment with mrTRG criteria, we have a powerful tool for tumor assessment after neoadjuvant treatment. Some studies conclude that there is a discrepancy between pCR and mrTRG [44,45]; however, Battersby et al. [46] in the clinical trial TRIGGER suggest that mrTRG1-2 correspond to states of non-tumor viability, and although they cannot be correlated with TRG they do correlate with complete clinical responses and are patients who can enter the WW program, representing the most substantial evidence in this context.

There is no question that MRI plays a crucial role in the pre- and post-treatment evaluation of neoadjuvant rectal cancer, notwithstanding certain limitations in assessment, such as fibrosis, inflammation, and edema. However, a notable lack of standardization exists regarding its use as a prognostic marker. This issue is expected to be addressed with the integration of radiomics and radiogenomics, which will facilitate the determination of pathological complete response (pCR) via MRI. Ultimately, these advancements will require validation through prospective studies to substantiate their clinical application [47] (Table 1).

## 4. Hematological Biomarkers in Rectal Cancer

### 4.1. Neutrophil–Lymphocyte Ratio (NLR)

It is considered the most relevant hematologic marker [48], defined as the ratio of the absolute neutrophil count to the absolute lymphocyte count in a single blood sample. Walsh et al. [49] first reported its prognostic significance in colorectal cancer (CRC), a finding subsequently supported by additional studies [49,50,51,52]. More recent investigations have further indicated a potential association with neoadjuvant treatment outcomes in locally advanced rectal cancer (LARC), as well as with pathological response rates [53,54,55]. However, this conclusion is based on studies utilizing diverse methodologies, predominantly retrospective in design, with varying ratio values and markedly heterogeneous populations. These factors have impeded its widespread application in routine clinical practice and hindered its standardization as a reliable predictive biomarker.

Prospective studies have contradictory conclusions on the prognostic value of the NLR. Novin et al. [48], after analyzing 86 patients with LARC, found a pCR of 23.3%; however, they did not find a correlation between the pre-neoadjuvant NLR and pCR, using ROC curves as a cut-off value. Gawinski et al. [56] evaluated 60 patients with LARC with non-significant data regarding NLR. On the other hand, Ergen et al. [57], with 53 patients and ROC curves as cut-off values, found a significant association between NLR and OS. Lino-Silva et al. [58], with a follow-up of 33.5 months and a sample of 175 patients, did not find a significant association between NLR and OS or with pCR. Polk et al. [59] correlated high NLR values with survival in patients with single metastasis of rectal cancer, obtaining significant data for OS and relapse-free survival. Data in the literature are contradictory; therefore, another trend is given by the combination of hematologic markers together with MRI data, which seems to be able to predict more reliably the possibility of pCR in patients with LARC (Table 2). The most commonly used cut-off value for the neutrophil-to-lymphocyte ratio is less than 3, with patients exhibiting higher values being more likely to experience complications and increased mortality [55].

### 4.2. Preoperative Plasma Fibrinogen and Neutrophil–Lymphocyte Ratio (F-NLR Score)

The coagulation cascade plays an important role in tumor progression and metastatic disease [60], especially fibrinogen, where high values correlate with poor prognosis in different neoplasms [61,62,63] and poor response to treatment and worse survival in LARC [64,65]. Therefore, F-NLR, which combines NLR and fibrinogen, has emerged as a possible new prognostic marker with good acceptance in gastric tumors [66], esophageal tumors [67], and lung tumors [68]. In LARC, its usefulness has not been determined. Retrospective data are available, as presented by Sun et al. [60], with a sample of 317 patients, use of ROC curves for cutoff values and a nomogram to predict DFS, correlating with high F-NLR values and poorly differentiated tumors, nodal involvement, and advanced stages, concluding that this marker can predict recurrences in patients with LARC who receive neoadjuvant therapy. De Felice et al. [69] evaluated 58 patients with LARC, determined the value of F-NLR prior to neoadjuvant therapy, and found worse survival in patients with elevated F-NLR; this analysis included patients with anal tumors. There is a lack of robust data supporting the use of this biomarker in clinical practice. The available data are primarily derived from retrospective series with small sample sizes. Nevertheless, it appears that its combination with other factors may have the potential to predict tumor response.

### 4.3. Platelet-to-Lymphocyte Ratio (PLR)

The platelet–lymphocyte ratio (PLR) originates from the absolute division of platelets and lymphocytes. The most widely accepted normal cutoff value is <150. Its elevated value is associated with worse prognosis in gastric tumors, pancreatic cancer, and breast and bladder cancer [57]; however, like the other markers, its use has not been standardized, and its value in LARC has not been determined. Ergen et al. [57] determined a correlation between OS, DFS, and elevated PLR. Another study retrospectively analyzed 111 patients with LARC and correlated them with different hematologic markers, highlighting the correlation of PLR with pathologic tumor response (ypT) [57].

### 4.4. Monocytes–Lymphocyte Ratio (MLR)

Another biomarker associated with inflammation and linked to locally advanced rectal cancer (LARC) is the monocyte-to-lymphocyte ratio (MLR). The most commonly used cut-off value for this biomarker ranges between 0.1 and 0.4. In a meta-analysis by Portale et al. [70], which included 14 studies with 14,205 patients, a low MLR was found to be associated with overall survival (OS) and disease-free survival (DFS), suggesting it could serve as an optimal prognostic marker. Similarly, Hamid et al. [71], in their meta-analysis of 14 studies comprising 6683 patients, evaluated the impact of MLR before chemotherapy (CT) and its relationship with DFS and OS. They found an association with OS, DFS, and even pathological complete response (pCR). However, subgroup analysis revealed no such relationship in patients treated with surgery alone, suggesting that the prognostic value of this ratio may be more relevant to neoadjuvant CT-RT. This biomarker could potentially help categorize patients as high-risk before the initiation of oncologic treatments. In another study, Yamamoto et al. [72] explored the relationship between MLR and prognosis in patients with LARC and found that the combination of this ratio with ypN staging could predict recurrences in patients undergoing neoadjuvant therapy. This further emphasizes the important role of neoadjuvant therapy in the utility of MLR, which appears to be one of the most robust biomarkers in LARC.

### 4.5. Inflammation Biomarkers

Inflammation in the tumor microenvironment can affect tumor proliferation, invasion, and development [73], hence the importance of multiple studies that have been dedicated to assessing the importance of these parameters, such as systemic immune-inflammation index (SII), prognostic nutritional index (PNI), and systemic inflammation response index (SIRI) [74]. The results so far are contradictory in the absence of well-elaborated studies that can determine its implication as a prognostic value in LARC. Chen et al. [75] reported that SII was the most effective prognostic factor for survival after surgery. On the contrary, Qi et al. [76] indicate that SIRI is the best prognostic indicator in different tumors, in agreement with previous studies where high values are associated with poor clinical outcomes [77].

Ding et al. [74], in their study, determined that SIRI and SII are a clear predictor of efficacy and prognosis of neoadjuvant treatment in LARC after uni- and multivariable analysis. On the other hand, Emrah Eraslan et al. [78] concluded in their article that SII may be a predictive factor for pCR in patients receiving neoadjuvant. However, Sun et al. [79], in their retrospective analysis, indicate that a combination of SII before and after surgery increases its predictive efficacy more than its baseline measurement at the beginning of treatment.

Zhang et al. [80] assessed 472 patients with LARC and constructed a nomogram with the most representative inflammatory markers. ISS did not show a relationship with OS, but other markers, such as NLR, were positive. On the other hand, Sun J et al. [81] constructed another nomogram where they included TNM, perineural invasion, an NLR, ISS, and Δpan-immune-inflammation value (ΔPIV), which, according to their data, can predict the risk of DFS at three years in patients with LARC submitted to radical treatment.

Inflammatory markers seem to be good predictors of response to neoadjuvant treatment, and even more, the combination of nomograms with other indicators, including radiological information, to predict in a better way the course of the disease. This can clearly have an impact on the adaptation of oncologic treatment in order to reduce toxicities and, in some cases, avoid mutilating surgeries.

### 4.6. Geriatric Patients and Inflammation Biomarkers

The patient’s condition prior to oncologic treatments predicts the tolerance and toxicity of CT and/or CT-RT, so early intervention would benefit outcomes in geriatric patients [82]. Ide S et al. [82] used the geriatric nutritional risk index (GNRI) formula (1.489 × albumin, g/L) + (41.7 × current weight/ideal weight) and, according to the cutoff value divided patients into two groups, with patients with low GNRI having worse OS and DFS. Adjuvant therapy apparently did not impact prognosis regardless of group. Therefore, the authors conclude that it is a useful tool to identify recurrence in geriatric patients with LARC. Minami S et al. [83] assessed the role of GNRI in 57 patients with LARC, determining postoperative complications and prognostic role, finding that patients with low GNRI have worse OS, this group of patients also had venous invasion and lymphatic invasion. Similar results have been published by Siyi Lu et al. [84] after analysis of 172 patients ≥ 60 years undergoing neoadjuvant therapy. Other parameters evaluated in patients ≥ 65 years by B Manoglu et al. [85] are the modified Glasgow Prognostic Score (mGPS) and the C-reactive protein–albumin ratio (CAR) and related to morbidity, mortality, local and distant recurrence, and OS, uni- and multivariate analysis determined that mGPS and CAR are independent prognostic factors of OS and can predict severe postoperative complications in both geriatric and non-geriatric populations.

GNRI, CAR, and mGPS are important parameters that show some robustness in predicting prognosis in geriatric patients; however, comprehensive geriatric assessment (CGA) remains one of the most distinctive elements in the selection of patients undergoing radical oncology treatments, Wen-Yang Liu et al. [86], determined in their phase II clinical trial that in patients considered fit through CGA the standard LARC treatment can be applied to be well tolerated and with similar efficacy to the rest of the population, which will undoubtedly impact the prognosis of these patients.

## 5. Extramural Vascular Invasion (EMVI) and Hematological Biomarkers

MRI-determined EMVI is a widely accepted poor prognostic factor [87], but hematologic markers are not, where data from different studies show discrepancy; however, a combination of both markers seems feasible to increase their prognostic probability. Gawinski et al. [87] performed a retrospective analysis that included a multivariate analysis of EMVI and its correlation with levels of neutrophils, lymphocytes, monocytes, platelets, and carcinoembryonic antigen (CEA), these hematologic markers were higher in patients with positive EMVI compared to the EMVI-negative cohort; however, the correlation of MRL, NLR, and PLR with EMVI did not prove to be a good predictor of response after neoadjuvant therapy [87].

## 6. Molecular: ctDNA

Circulating tumor DNA (ctDNA) is obtained through liquid biopsy, which is derived from apoptotic cells, necrosis, or exosomes [88,89], representing <1% of circulating DNA [90]. This marker in preliminary studies may serve to monitor tumor response, tumor recurrences, and treatment resistance [91,92,93]. However, the low concentrations of ctDNA in plasma, inflammation, trauma, and manipulation during analysis decrease the sensitivity of its detection, and it is not possible to determine the invasiveness of the tumor or its heterogeneity. However, despite the limitations, it is more sensitive than CEA or imaging tests [94]. Therefore, its use as a biomarker to predict response to neoadjuvant treatment in LARC is nowadays a matter of study [91,93,95,96,97,98,99]. Morais et al. [94] assessed the correlation between ctDNA and pCR; their results found no association between ctDNA and pCR as found in other studies [95,96], however, new mutations were found through next-generation sequencing NGS possibly induced by neoadjuvant treatment such as MAF, although according to this study it does not predict response to treatment.

Chang et al. [100], in their meta-analysis, combined ctDNA data with OS, finding that ctDNA after CT-RT and surgery is associated with the risk of recurrence, especially ctDNA after CT-RT, is associated with OS. Khakoo et al. [101] objectified that ctDNA detection after surgery is associated with worse OS. On the other hand, ctDNA detection is not associated with the probability of acquiring pCR [100], this is related to the fact that ctDNA diagnoses disease, including minimal residual disease (MRD), and cannot predict the probability of obtaining pCR after surgery [95].

## 7. Tumor Budding

Tumor budding has been associated with a worse prognosis in patients with LARC, related to the loss of tumor cell adhesion, which is an “indication” of the onset of the metastatic process [102]. According to the International Tumor Budding Consensus Conference (ITBCC) [103], the classification is as follows:

- BD1: 0–4 buds, low budding.
- BD2: 5–9 buds, intermediate budding.
- BD3: ≥10 buds, high budding.

Both BD2 and BD3 are risk factors for lymph node metastases in stage I (pT1) patients. Patients with stage II BD3 are candidates for adjuvant therapy [102].

## 8. Specific Markers of Radiotherapy Response

Personalization of cancer treatment leads to the search for radiotherapy-specific response markers. Some studies report that the MRE11/RAD50/NBS1 (MRN) protein complex pathway involved in DNA detection and repair may play an important role since its expression appears to be significantly associated with OS and DFS in patients treated with neoadjuvant treatment and may even help to identify radiosensitive tumors [102,104]. Another scenario under study involves the addition of poly-ADP ribose polymerase (PARP) inhibitors since radiotherapy appears to potentiate the effect of PI3K and PARP inhibitors [105]. Carcinoembryonic antigen (CEA) has been shown to be a predictor that acts independently of neoadjuvant treatment in LARC [102].

## 9. Microsatellite Instability (MSI)

During DNA replication, the mismatch repair (MMR) system, comprising the MLH1, MSH2, MSH3, MSH6, and PMS2 proteins, is responsible for correcting errors that arise during the replication process [106]. Deficiencies in any of these proteins lead to replication errors, particularly within microsatellites, a condition known as microsatellite instability (MSI), which is associated with an elevated risk of carcinogenesis, including CRC [106]. The detection of MSI is critical for guiding treatment decisions, and it is recommended to assess it in all patients. De Rosa et al. [107] found that patients with deficient mismatch repair (dMMR) exhibited improved prognostic outcomes, and recent studies suggest that reducing the intensity of treatment may enhance oncological results in these patients [108,109,110]. These individuals demonstrate heightened sensitivity to immunotherapy, with responses that are often durable [111,112], and they experience higher rates of pCR without the need for CT-RT or surgery [113,114,115]. The NCCN Guidelines recommend checkpoint inhibitor immunotherapy for up to 6 months in patients with dMMR [116], and in the event of a complete clinical response, surveillance without surgical intervention is advised. Consequently, the determination of MSI is essential in all patients to ensure appropriate treatment tailoring.

An important consideration is the cost-effectiveness of these biomarkers. To date, there is a lack of studies specifically addressing this issue, with most research focusing on the use of biomarkers such as CEA for monitoring and assessing relapse. Some studies have reported costs associated with the detection of local relapse via CEA ranging from USD 10,446 to USD 24,779 [117], while a German study [118] estimates the cost at EUR 4851. It is essential to determine the cost-effective value of biomarkers in the neoadjuvant setting, particularly those that more accurately predict the pathological complete response (pCR) and potentially reduce the need for surgery, hospital admissions, and complications associated with surgical interventions.

## 10. Perspectives Futures

The direction of rectal cancer treatment is increasingly focused on enhancing neoadjuvant therapies to achieve higher rates of pCR with reduced surgical intervention, underscoring the importance of identifying novel biomarkers to optimize treatment strategies. One emerging field is radiomics, which provides non-visual information by extracting numerous quantitative features from medical imaging. Its application in rectal cancer is promising. Shin et al. [31] evaluated an MRI radiomics model combining T2-weighted imaging and diffusion-weighted imaging (DWI) to predict pCR following chemotherapy–radiotherapy (CT-RT) in 898 patients with locally advanced rectal cancer (LARC) after neoadjuvant therapy (NAT). The study found that the pCR rate was 21%, with merged models demonstrating no significant advantage over T2-weighted models, while DWI performed worse than the merged models, opening new possibilities for radiomics models. A similar conclusion was reached by Miranda et al. [119] in their narrative review, which highlighted that radiomics has the potential to provide valuable insights for clinical decision-making. However, challenges remain, particularly in the standardization of imaging protocols, feature extraction, and model validation. Other models under investigation include spatiotemporal multi-omics analysis using artificial intelligence and machine learning, which is being tested in three prospective trials [120]. Additionally, the inclusion of anti-EGFR and anti-angiogenic agents in the neoadjuvant setting is also a promising avenue [120], as is the development of patient-derived tumor organoids (PDTOs) that may predict responses to chemotherapy and radiotherapy [121].

## 11. Conclusions

Neoadjuvant therapy in locally advanced rectal cancer (LARC) has seen significant advancements in recent years, particularly with the introduction of TNT. This approach has resulted in higher rates of pCR compared to those observed in earlier studies. However, it remains to be determined which patient subgroups are most likely to achieve pCR or exhibit better prognostic outcomes in order to facilitate treatment de-escalation or personalized treatment strategies. Current evidence suggests that no single biomarker is capable of reliably predicting disease progression. Instead, the combination of multiple biomarkers appears to be the key. In this regard, post-neoadjuvant magnetic resonance imaging (MRI) functional sequences, alongside specific hematological biomarkers, stand out, with the monocyte-to-lymphocyte ratio (MLR) emerging as one of the most relevant in this context. Additionally, the combination of MRI with circulating tumor DNA (ctDNA) detection shows promise in identifying and characterizing minimal residual disease (MRD). Inflammatory markers, which integrate several parameters, have proven to be independent prognostic indicators. Furthermore, the application of these biomarkers in the geriatric population presents a valuable opportunity to personalize treatments and improve prognostic assessment in these patients. Despite these advancements, there remains a need to standardize biomarkers, establish consensus cut-off values, and validate their clinical utility in randomized trials to enable their routine incorporation into clinical practice

## Figures and Tables

**Table 1:** Biomarkers in rectal cancer.

Biomarker	Specific Subtype
Imaging: Magnetic Resonance Imaging (MRI)	Diffusion-weighted imaging (DWI) Apparent diffusion coefficient (ADC) Perfusion
Haemathological	Neutrophil–Lymphocyte Ratio (NLR) Preoperative Plasma Fibrinogen and Neutrophil–Lymphocyte Ratio (F-NLR score) Platelet-to-Lymphocyte Ratio (PLR) Monocytes–Lymphocyte Ratio (MLR)
Inflammatory	Systemic immune-inflammation index (SII) Prognostic nutritional index (PNI) Systemic inflammation response index (SIRI) Δpan-immune-inflammation value (ΔPIV)
Geriatric population	Geriatric nutritional risk index (GNRI) Glasgow Prognostic Score (mGPS) C-reactive protein–albumin ratio (CAR)
Molecular	ctDNA
Pathologic	Extramural Vascular Invasion (EMVI) Tumor Budding Microsatellite instability (MSI)

**Table 2:** Ongoing studies with prognostic markers in rectal cancer.

Study Name	Type of Study	Intervention	Objective
Prognostic Value of Neutrophil-to-lymphocyte Ratio (NLR) on Rectal Cancer Patients (NCT03015168)	Retrospective	Patients with rectal cancer undergoing capecitabine and concurrent intensity modulated radiotherapy	- OS* - Grade 3 or higher treatment related small bowel toxicity
The Prognostic Impact of The Neutrophil-to-Lymphocyte Ratio In Patients With Locally Advanced Rectal Cancer (NCT05673343)	Cohort Prospective	Blood test 2 weeks before CT-RT*** - CT-RT (TNT****) + Surgery - CT-RT + Surgery + CT	- DFS** - OS
Neutrophil-to-Lymphocyte Ratio (NLR) and C-reactive Protein (CRP) as New Markers in Diagnosis and Prediction of Colorectal Cancer (NelyCre) (NCT05129046)	Prospective	- Neutrophil–Lymphocyte Ratio (NLR) - C-Reactive Protein (CRP)	- DFS - OS - Surgical results (postoperative complications and changes in the NLR y CRP)
Preoperative Nutritional Status and Postoperative Outcomes in Colorectal Cancer (NCT06016829)	Prospective	Test application: - Nutrition Risk Screening 2002 (NRS-2002) - Global Leadership Initiative on Malnutrition (GLIM) - Measurement of body composition with computed tomography (CT) - Dietary inflammatory index (DII) - EORTC-QLQ-CR29 - Prognostic Nutritional Index (PNI) - NLR	- Postoperative complications - Postoperative quality of life - EORTC-QLQ-CR29
Advanced MR Imaging for Early Biologic Tumor Changes to Neoadjuvant Chemoradiation Treatment for Rectal Cancer (NCT01830582)	Prospective Randomized	Experimental 1A: Diagnostic MRI*****. MRI 24 h after 1 session of RT. MRI during 2 weeks of RT. MRI after end of RT prior surgery. Experimental 1B: Diagnostic MRI. MRI 48 h after 1 session of RT. MRI during 3 weeks of RT. MRI after end of RT prior surgery. Experimental 1C: Diagnostic MRI. MRI 72 h after 1 session of RT. MRI during 4 weeks of RT. MRI after end of RT prior surgery. Experimental 2: Diagnostic MRI. MRI 24, 48 or 72 h after 1 session of RT. MRI during 2, 3 or 4 weeks of RT. MRI after end of RT prior surgery.	- Determine the best scheme for obtaining MRI. - Determine whether the best imaging scheme is DWI and DCE.
PET/ MRI as a Predictor of Response to Pre-op Chemoradiation in Resectable Rectal Cancer: a Pilot Study (NCT01751516)	Prospective	PET/MRI scans before and after surgery	- Negative post-CT-RT PET/MRI scan, as correlated with surgical pathology. - Recurrence free survival - Disease-specific survival - OS
Predicting RadIotherapy ReSponse of Rectal Cancer With MRI and PET (PRISM) (NCT02233374)	Prospective Randomized	Early MRI and PET/CT 2 weeks after commencing chemo/RT’ involves additional Multiparametric MRI + PET/CT 2 weeks into chemo/RT Intervention Late MRI and PET/CT 6 weeks post chemo/RT’ involves additional Multiparametric MRI + PET/CT 6 weeks post chemo/RT	- Predictive value of PET/CT and MRI 2 weeks into chemo-irradiation for developing a pathologic complete response at surgery. - Feasibility of conducting additional PET and MRI scans at 6 weeks post-treatment. - Utility of adding PET scan to the baseline staging of patients. - Pathologic response according to Tumor Regression Grade (TRG). - Impact of pCR rates on long term disease control
Predicting Neoadjuvant Therapy Response of Rectal Cancer With MRI (NCT02640586)	Prospective	Three MR examinations: - First MRI taken within 1 week before preoperative chemo-radiotherapy. - Second MRI taken between 14–16 days after the initiation of radio-chemotherapy. - Third MRI taken 7–9 weeks after the completion of preoperative chemo-radiotherapy.	- Predictive value of early MRI (14–16 days MRI) for assessing pathological complete response(pCR) at surgery. - Predictive value of preoperative MRI(7–9 weeks after the completion of preoperative chemo-radiotherapy) for assessing pCR******.

OS*: Overall survival. DFS**: disease-free survival. CT-RT***: chemoradiation. TNT****: total neoadjuvant therapy. MRI*****: magnetic resonance image. pCR******: pathological complete response.
